# Adherence to Healthy Lifestyle Prior to Infection and Risk of Post–COVID-19 Condition

**DOI:** 10.1001/jamainternmed.2022.6555

**Published:** 2023-02-06

**Authors:** Siwen Wang, Yanping Li, Yiyang Yue, Changzheng Yuan, Jae Hee Kang, Jorge E. Chavarro, Shilpa N. Bhupathiraju, Andrea L. Roberts

**Affiliations:** 1 Department of Nutrition, Harvard T.H. Chan School of Public Health, Boston, Massachusetts; 2 Zhejiang University School of Public Health, Hangzhou, China; 3 Channing Division of Network Medicine, Department of Medicine, Brigham and Women’s Hospital and Harvard Medical School, Boston, Massachusetts; 4 Department of Epidemiology, Harvard T.H. Chan School of Public Health, Boston, Massachusetts; 5 Department of Environmental Health, Harvard T.H. Chan School of Public Health, Boston, Massachusetts

## Abstract

**Importance:**

Few modifiable risk factors for post–COVID-19 condition (PCC) have been identified.

**Objective:**

To investigate the association between healthy lifestyle factors prior to SARS-CoV-2 infection and risk of PCC.

**Design, Setting, and Participants:**

In this prospective cohort study, 32 249 women in the Nurses’ Health Study II cohort reported preinfection lifestyle habits in 2015 and 2017. Healthy lifestyle factors included healthy body mass index (BMI, 18.5-24.9; calculated as weight in kilograms divided by height in meters squared), never smoking, at least 150 minutes per week of moderate to vigorous physical activity, moderate alcohol intake (5 to 15 g/d), high diet quality (upper 40% of Alternate Healthy Eating Index–2010 score), and adequate sleep (7 to 9 h/d).

**Main Outcomes and Measures:**

SARS-CoV-2 infection (confirmed by test) and PCC (at least 4 weeks of symptoms) were self-reported on 7 periodic surveys administered from April 2020 to November 2021. Among participants with SARS-CoV-2 infection, the relative risk (RR) of PCC in association with the number of healthy lifestyle factors (0 to 6) was estimated using Poisson regression and adjusting for demographic factors and comorbidities.

**Results:**

A total of 1981 women with a positive SARS-CoV-2 test over 19 months of follow-up were documented. Among those participants, mean age was 64.7 years (SD, 4.6; range, 55-75); 97.4% (n = 1929) were White; and 42.8% (n = 848) were active health care workers. Among these, 871 (44.0%) developed PCC. Healthy lifestyle was associated with lower risk of PCC in a dose-dependent manner. Compared with women without any healthy lifestyle factors, those with 5 to 6 had 49% lower risk (RR, 0.51; 95% CI, 0.33-0.78) of PCC. In a model mutually adjusted for all lifestyle factors, BMI and sleep were independently associated with risk of PCC (BMI, 18.5-24.9 vs others, RR, 0.85; 95% CI, 0.73-1.00, *P* = .046; sleep, 7-9 h/d vs others, RR, 0.83; 95% CI, 0.72-0.95, *P* = .008). If these associations were causal, 36.0% of PCC cases would have been prevented if all participants had 5 to 6 healthy lifestyle factors (population attributable risk percentage, 36.0%; 95% CI, 14.1%-52.7%). Results were comparable when PCC was defined as symptoms of at least 2-month duration or having ongoing symptoms at the time of PCC assessment.

**Conclusions and Relevance:**

In this prospective cohort study, pre-infection healthy lifestyle was associated with a substantially lower risk of PCC. Future research should investigate whether lifestyle interventions may reduce risk of developing PCC or mitigate symptoms among individuals with PCC or possibly other postinfection syndromes.

## Key Points

**Question:**

Is a healthy lifestyle (healthy body mass index, never smoking, high-quality diet, moderate alcohol intake, regular exercise, and adequate sleep) prior to SARS-CoV-2 infection protective of post–COVID-19 condition (PCC)?

**Findings:**

In this prospective cohort study of 1981 women who reported a positive SARS-CoV-2 test from April 2020 to November 2021, adherence to a healthy lifestyle prior to infection was inversely associated with risk of PCC in a dose-dependent manner. Compared with those who did not have any healthy lifestyle factors, those with 5 or 6 had half the risk of PCC.

**Meaning:**

Preinfection healthy lifestyle was associated with a substantially decreased risk of PCC.

## Teaser

This prospective cohort study investigates the association between healthy lifestyle factors prior to SARS-CoV-2 infection and risk of post–COVID-19 condition.

## Introduction

*Post–COVID-19 condition *(PCC), informally known as *long COVID*, is defined as having COVID-19 symptoms for at least 4 weeks after initial SARS-CoV-2 infection.^1^ This condition is estimated to affect 20% to 40% of individuals with COVID-19.^2,3^ The prevalence is higher among persons who were not vaccinated against COVID-19 or who were hospitalized for COVID-19 during the acute phase, reaching 50% to 70%.^4,5,6^ The condition has a wide range of respiratory, cardiovascular, metabolic, gastrointestinal, neurological, and psychiatric manifestations, which can influence daily functioning.^7^ With ongoing waves of SARS-CoV-2 infection, PCC has created a serious public health burden, with an estimated 8 to 23 million Americans having developed PCC.^8^ Thus, better understanding of PCC causes is critical.

Persistent inflammation has been implicated in PCC symptoms related to multiple organs.^9,10^ Inflammatory factors have also been associated with other postinfection syndromes, such as postviral fatigue syndrome.^11,12^ Healthy lifestyle factors, including healthy body mass index (BMI; calculated as weight in kilograms divided by height in meters squared),^13^ abstinence from cigarette smoking,^14^ a healthy diet,^15^ moderate alcohol consumption,^16^ regular exercise,^17^ and adequate sleep,^18^ have been identified as protective against inflammation. Adherence to multiple healthy lifestyle factors is associated with less severe COVID-19 disease as well as lower mortality from infectious diseases (including COVID-19), in a dose-dependent manner.^19,20^

Prior studies have found healthy BMI was associated with lower risk of PCC and inconsistent associations between smoking and PCC.^21,22,23^ The association between multiple healthy lifestyle factors prior to infection and risk of PCC has not been established.

In this prospective cohort study, we investigated the association of adherence to modifiable risk factors prior to infection (eg, healthy BMI, never smoking, healthy diet, moderate alcohol consumption, regular exercise, and adequate sleep) with the risk of developing PCC among participants subsequently infected with SARS-CoV-2. We further examined the extent to which established risk factors for COVID-19 severity (eg, hypertension, asthma)^21,24^ might account for possible associations. In addition, among individuals with PCC, we explored whether preinfection healthy lifestyle was associated with number of PCC symptoms and PCC-related daily life impairment.

## Methods

Participants were from an ongoing longitudinal cohort, the Nurses’ Health Study II, which in 1989 enrolled 116 429 female nurses residing in the US aged 25 to 42 years.^25^ Biennial follow-up questionnaires are sent to query lifestyle characteristics and health. Response rates exceeded 85% at each follow-up cycle. In April 2020 (termed *baseline* henceforth), a COVID-19 substudy invitation was sent to active cohort participants to assess health during the pandemic, with monthly and quarterly follow-up surveys administered through November 2021 (55 925 invited, 39 137 [71%] responded) (eFigure 1 in the Supplement).

Of women who responded to the COVID-19 substudy baseline and final questionnaires, 32 249 women had returned the 2017 biennial questionnaire querying lifestyle factors. During 19 months of follow-up, 2303 participants (7.1%) reported a positive SARS-CoV-2 test (antibody, antigen, or PCR [polymerase chain reaction]) and the date of that test. In main analyses, we excluded 89 participants who did not have complete information about lifestyle factors and 233 participants who did not answer the PCC question, leaving 1981 participants (eFigure 2 in the Supplement).

The study was approved by the Brigham and Women's Hospital institutional review board. Return of questionnaires implied informed consent. Results are reported in accordance with the Strengthening the Reporting of Observational Studies in Epidemiology (STROBE) reporting guideline.

### Assessment of Healthy Lifestyle

Six potentially modifiable lifestyle factors were assessed, including BMI, smoking, alcohol consumption, diet, physical activity, and sleep (2015 for diet and alcohol intake, 2017 for others). Self-report of weight and height has been validated in this cohort.^26^ Smoking was queried every 2 years, and we characterized lifetime smoking history as never, past, or current smoking. In a validation study, toenail nicotine level was strongly associated with reported smoking level (Spearman *r*, 0.63).^27^ Diet in the past year was measured using a validated semiquantitative food frequency questionnaire (FFQ).^28,29^ To characterize overall diet quality, we used the Alternative Healthy Eating Index (AHEI-2010), which is based on empirical evidence^30^ (higher score indicates healthier diet), excluding the alcohol component. Alcoholic beverage consumption was also collected by the FFQ. Physical activity was assessed using a validated questionnaire.^31^ For each participant, we estimated the average time spent in the past year in moderate to vigorous recreational activities (eg, running, jogging, cycling, tennis, squash, racquet ball, swimming, weight or resistance training, brisk walking, and other vigorous activities). We queried average sleep in a 24-hour period, with response options ranging from less than 5 to at least 10 hours. Daily sleep duration has been validated.^32^

### Healthy Lifestyle Score

We defined 6 healthy lifestyle factors as healthy body weight (BMI, 18.5-24.9), never smoking, at least 150 minutes per week of moderate to vigorous physical activity, high diet quality (upper 40% of AHEI-2010 score), moderate alcohol intake (5-15 g/d), and adequate sleep (7-9 h/d), in accordance with US government recommendations or prior evidence.^33,34,35,36^

For each of the 6 factors, we created a binary variable, with participants receiving a score of 1 if they met the criteria for healthy and 0 otherwise. We then calculated the total number of healthy lifestyle factors for each participant. Because only 36 women had all 6 healthy lifestyle factors, women with 5 or 6 factors were combined in analyses.

### SARS-CoV-2 Infection and PCC

SARS-CoV-2 infection and PCC ascertainment in this cohort has been described elsewhere.^37^ Briefly, past 7-day, 30-day, and 90-day positive SARS-CoV-2 test (antibody, antigen, or PCR) and hospitalization due to COVID-19 since March 1, 2020, were self-reported on each follow-up questionnaire in the COVID-19 substudy. Post–COVID-19 condition was assessed on the final substudy questionnaire, administered 12 months after baseline. Participants were asked, “Have you experienced any long-term COVID-19 symptoms (lasting for more than 4 weeks)?” If yes, participants were asked to indicate which symptoms they experienced (eMethods in the Supplement). Participants with self-reported PCC were asked: (1) whether symptoms were ongoing; (2) duration of symptoms (less than 2 months; 2-3 months; 4-5 months); and (3) how often the symptoms prevented them from carrying out daily activities.

### Covariates

Date of birth (ascertained in 1989), racial and ethnic group (1989), and partner’s educational attainment (1999) were self-reported. Census tract median income and percentage with bachelor’s degree or higher were assessed based on geocoded residence in 2009. Lifetime history of physician-diagnosed diseases has been updated biennially (eMethods in the Supplement). Frontline health care worker status was self-reported at COVID-19 substudy baseline. COVID-19 vaccination status and date of vaccination were assessed on quarterly follow-up questionnaires.

### Statistical Analysis

Among participants who reported a positive SARS-CoV-2 test over follow-up, we first compared sociodemographic factors and distribution of preinfection healthy lifestyle factors among those who responded to the PCC question vs those who did not. We then compared the prevalence of sociodemographic and lifestyle factors by the number of healthy lifestyle factors. Each variable was missing less than 5%. Indicator variables were used for any missing covariate information for categorical variables; no participants were missing data for continuous variables.

We estimated the relative risks (RRs) and 95% CIs for the associations between the healthy lifestyle score and risk of PCC, adjusting for age, race and ethnicity, partners’ education, census tract median household income, census tract percentage population with bachelor’s degree or higher, health care worker status, and history of chronic diseases (fully adjusted model) using Poisson regression. We also estimated the RRs and 95% CIs for the associations between individual healthy lifestyle factors (both as categorical/continuous variables and dichotomized) with risk of PCC. We further fit models mutually adjusted for all healthy lifestyle factors. In addition, we calculated the population attributable risk percentage (PAR). PAR estimates the proportion of PCC in this cohort that hypothetically would not have occurred if effect estimates reflected causal relationships and all participants were in the low-risk group.^38^ To calculate the PAR, we used RRs and 95% CIs from the fully adjusted model including all 6 healthy lifestyle factors.

To estimate the PAR in the US population, we used the prevalence of the 6 lifestyle factors among women of the same age as our study (ages 55-75 years) in the nationally representative US National Health and Nutrition Examination Survey (NHANES, 2013-2014). Further, among persons who developed PCC, we compared frequency of PCC symptoms and daily-life impairment due to PCC by healthy lifestyle score.

We conducted 10 sensitivity analyses. First, we defined PCC as having symptoms lasting for more than 2 months and more than 4 months. Second, as not all participants may have had access to testing, we expanded the definition of SARS-CoV-2 infection to include participants having symptoms without a confirmed test (926 COVID-19 cases were added). Third, to investigate whether the observed associations were explained by the severity of acute phase disease, we excluded participants who had been hospitalized due to COVID-19. Fourth, we used multiple imputation for missing healthy lifestyle (n = 89) or PCC (n = 233) information. Fifth, to reduce possible recall bias, we restricted PCC cases to 633 participants who reported ongoing symptoms at the time of PCC assessment. Sixth, to distinguish PCC symptoms from symptoms related to sleep deprivation, we excluded participants reporting only psychological, cognitive, or neurological symptoms. Seventh, we excluded 497 persons reporting fatigue as one of their long-term symptoms (among whom 48 reported fatigue as their only symptom). Eighth, because risk of PCC may be reduced among those who were vaccinated against COVID-19, we additionally adjusted for vaccination status at time of infection.^6^ Ninth, as low-to-moderate vs no alcohol consumption has been associated with both better and worse health,^39,40^ we excluded alcohol from the healthy lifestyle score. Tenth, we investigated whether observed associations differed by health care worker status by adding a cross-product term to the model. All analyses were conducted in SAS statistical software, version 9.4 (SAS Institute). All statistical tests were 2-sided. Significance level was assessed at *P* < .05.

## Results

The mean (SD) age of 32 249 participants was 65.9 (4.5) years (range 55-75 years). Of those, 97.2% were White, and 28.7% were active health care workers. Participants missing PCC data (n = 233) (vs those nonmissing [n = 1981]) were more likely to be racial and ethnic minorities, have lower BMI, be health care workers, have higher socioeconomic status, sleep less, and be less likely to have type 2 diabetes (eTable 1 in the Supplement). We documented 1981 participants with a positive SARS-CoV-2 test over 19 months of follow-up. Among those participants, mean age was 64.7 years (SD, 4.6; range, 55-75); 97.4% (n = 1929) were White; and 42.8% (n = 848) were active health care workers. The median time from assessment of exposures (return of 2017 questionnaire) to SARS-CoV-2 infection was 35 months (IQR, 31 months-37 months).

Healthy lifestyle factors were weakly to moderately correlated with each other (Φ coefficient range, −0.03 to 0.24; eTable 2 in the Supplement). At baseline, those who had greater healthy lifestyle scores were younger, more likely to be White, had higher socioeconomic status, and had lower prevalence of comorbidities (Table 1). Of participants who reported a positive SARS-CoV-2 test during follow-up, 44.0% (n = 871) reported PCC. Among these, 87.0% (n = 758) reported symptoms lasting at least 2 months, and 56.5% (n = 491) reported at least occasional daily life impairment related to PCC. The most common symptoms were fatigue (57.1%, n = 497), smell or taste problems (40.9%, n = 356), shortness of breath (25.3%, n = 220), confusion/disorientation/brain fog (21.6%, n = 188), and memory issues (20.0%, n = 174).

**Table 1.** Age-Standardized Participant Characteristics by Number of Healthy Lifestyle Factors Prior to the Pandemic Among Participants Who Reported a Positive SARS-CoV-2 Test During Follow-up, the Nurses’ Health Study II, 2015-2021^a^

Characteristic	No. (%) (n = 1981)					
	No. of healthy lifestyle factors					
	0 (n = 66)	1 (n = 301)	2 (n = 564)	3 (n = 518)	4 (n = 344)	5 or 6 (n = 188)
Age, mean (SD), y^b^	65.8 (4.3)	65.2 (4.5)	65.1 (4.6)	64.3 (4.7)	64.4 (4.4)	64.0 (4.7)
Race, White^c^	64 (96.8)	291 (96.8)	554 (98.2)	495 (95.5)	338 (98.3)	187 (99.5)
Partner’s education ≤ high school	14 (21.8)	60 (19.8)	108 (19.1)	77 (14.8)	37 (10.7)	13 (7.1)
Census tract median household income, mean (SD), USD	56 248 (17 550.8)	59 814.9 (19 479.1)	63 283.7 (22 513.7)	66 416.3 (25 742.1)	69 275.8 (26 415.7)	71 252.7 (26 143.9)
Census tract % population with bachelor’s degree or higher, mean (SD)	24.0 (14.9)	24.7 (13.8)	28.6 (15.9)	31.8 (17.8)	35.0 (17.7)	36.7 (18.6)
BMI, mean (SD)	32.5 (8.7)	32.6 (6.4)	30.2 (6.4)	27.3 (5.9)	25.3 (4.4)	22.8 (2.6)
AHEI, mean (SD)	52.1 (8.4)	53.4 (10)	56.8 (10.3)	62.1 (11.7)	66.7 (10.9)	72.6 (8.5)
Alcohol, mean (SD), g/d	4.0 (9.7)	6.2 (14)	6.7 (12)	7.9 (11.7)	7.2 (8.5)	9.4 (7.7)
Physical activity, min/wk	24.0 (33.0)	63.3 (153.9)	131 (258.9)	266.8 (318.5)	373.7 (339.5)	478 (402.2)
Sleep, h/d						
≤5	11 (16.7)	40 (13.3)	56 (10.0)	28 (5.5)	15 (4.2)	0 (0.0)
6	51 (77.5)	154 (51.0)	116 (20.6)	107 (20.6)	43 (12.4)	8 (4.0)
7	0 (0)	53 (17.7)	213 (37.8)	231 (44.6)	193 (56.2)	101 (53.8)
8	0 (0)	38 (12.7)	137 (24.4)	122 (23.5)	80 (23.3)	72 (38.4)
9	0 (0)	6 (2.1)	37 (6.6)	26 (5.0)	13 (3.8)	7 (3.7)
≥10	4 (5.8)	9 (3.1)	4 (0.7)	4 (0.8)	0 (0.0)	0 (0.0)
Smoking						
Never	0 (0.0)	129 (42.7)	360 (63.9)	367 (70.9)	280 (81.4)	175 (92.9)
Past	58 (87.6)	161 (53.6)	185 (32.9)	146 (28.3)	63 (18.3)	13 (7.1)
Current	8 (12.4)	11 (3.7)	18 (3.2)	4 (0.8)	1 (0.3)	0 (0.0)
Frontline health care worker^d^	26 (38.8)	138 (45.7)	259 (45.9)	221 (42.7)	135 (39.4)	73 (38.8)
Lifetime history of comorbidities						
High cholesterol	52 (79.4)	199 (66.1)	349 (61.8)	318 (61.4)	193 (56.1)	92 (49.1)
Diabetes	18 (27.4)	64 (21.2)	80 (14.3)	46 (8.9)	15 (4.3)	5 (2.4)
Hypertension	42 (64.3)	177 (58.7)	276 (48.9)	205 (39.6)	103 (30.1)	45 (23.9)
Asthma	23 (35.5)	83 (27.5)	144 (25.6)	112 (21.6)	56 (16.2)	33 (17.7)
Cancer	18 (28.0)	77 (25.4)	103 (18.3)	101 (19.4)	81 (23.4)	35 (18.7)
Chronic obstructive pulmonary disease	12 (18.7)	22 (7.3)	28 (5.0)	22 (4.2)	10 (2.9)	2 (1.3)
Cardiovascular disease^e^	12 (18.3)	45 (14.8)	50 (8.9)	53 (10.2)	17 (5.0)	2 (1.3)
Inflammatory bowel disease	2 (3.6)	8 (2.7)	21 (3.7)	13 (2.5)	9 (2.6)	2.6 (5)
Hospitalization due to COVID-19	8 (12.0)	23 (7.8)	36 (6.4)	21 (4.0)	6 (1.6)	5 (2.6)
COVID-19 vaccination (first dose) by the time of infection	6 (9.5)	15 (5.0)	38 (6.7)	35 (6.8)	24 (6.9)	7 (3.6)

Abbreviations: AHEI, Alternative Healthy Eating Index; BMI, body mass index (calculated as weight in kilograms divided by height in meters squared).

^a^ Healthy lifestyle factors include healthy body weight (BMI, 18.5-24.9), never smoking, at least 150 min/wk of moderate to vigorous physical activity, high diet quality (upper 40% of AHEI-2010 score), moderate alcohol intake (5-15 g/d), and adequate sleep (7-9 h/d). Diet and alcohol intake were assessed in 2015, all other lifestyle factors were assessed in 2017.

^b^ Not age-standardized.

^c^ Race and ethnicity were self-reported at cohort entry. Values for categories other than White (American Indian/Alaska Native, Asian, Black or African American, Native Hawaiian or Pacific Islander, and other) are not presented because their number were small.

^d^ Defined as physically working at a site providing clinical care.

^e^ Included angina, coronary artery bypass graft, myocardial infarction, stroke, and deep vein thrombosis.

**Figure 1.**
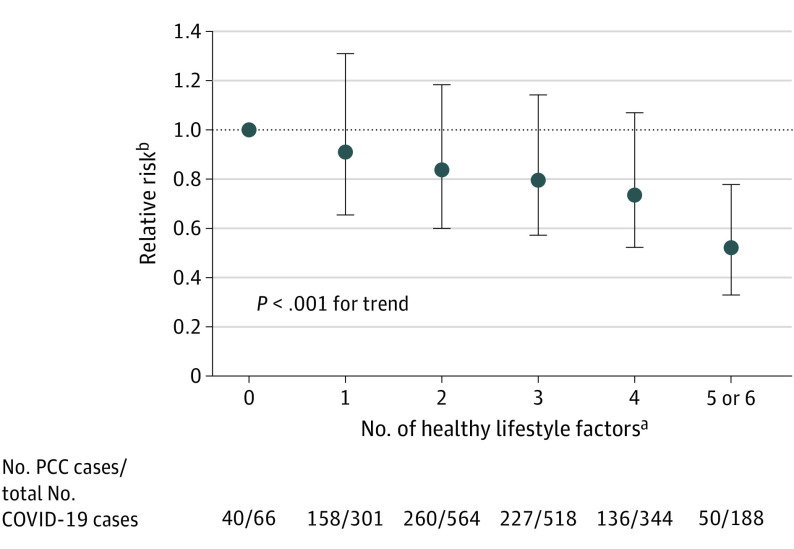
Number of Healthy Lifestyle Factors Prior to the Pandemic and Risk of Post–COVID-19 Condition (PCC) Among Participants Who Reported a Positive SARS-CoV-2 Test During Follow-up, the Nurses’ Health Study II, 2015-2021 *P* for trend analysis used indicator levels as a continuous variable. Error bars indicate 95% CIs. ^a^In the 1981 female participants, healthy lifestyle factors include healthy body weight (body mass index, 18.5-24.9; calculated as weight in kilograms divided by height in meters squared), never smoking, at least 150 min/wk of moderate to vigorous physical activity, high diet quality (upper 40% of Alternative Healthy Eating Index [AHEI]–2010 score), moderate alcohol intake (5-15 g/d), and adequate sleep (7-9 h/d). Diet and alcohol intake were assessed in 2015; all other lifestyle factors were assessed in 2017. ^b^Adjusted for age; race and ethnicity; health care worker status; partner’s education; census tract median household income; census tract percentage population with bachelor’s degree or higher; and history of chronic obstructive pulmonary disease, cancer, diabetes, asthma, hypertension, high cholesterol, cardiovascular diseases, and inflammatory bowel disease.

Risk of PCC was lower with increasing number of healthy lifestyle factors (Figure 1; *P* < .001 for trend). Compared with women who did not adhere to any healthy lifestyle factors, those having 5 or 6 factors had a 49% lower risk of PCC (RR, 0.51; 95% CI, 0.33-0.78, Figure 1). Assuming a causal relationship, the PAR for healthy lifestyle was 36.0% (95% CI, 14.1%-52.7%).

In analyses examining each healthy lifestyle factor separately, BMI, smoking, diet, and physical activity were associated with risk of PCC in models adjusting for demographic factors (Table 2, Model 1). Compared to low or high levels, moderate alcohol consumption and 7 to 9 h/d sleep had the lowest risk of PCC (Table 2, Model 1). When further adjusted for socioeconomic status, health care worker status, and comorbidities, the associations were slightly attenuated (Table 2, Model 2). When we categorized the 6 lifestyle factors as binary variables, BMI and sleep were independently associated with risk of PCC in models mutually adjusted for all factors (Table 2, Model 3; Table 3). If these relationships were causal, the PAR (95% CI) for overweight/obesity was 10.3% (95% CI, 0.2%-19.8%) and for inadequate sleep was 6.6% (95% CI, 1.8%-11.5%, Table 3). PARs for other healthy lifestyle factors ranged from 2.4% to 4.5%. Results were comparable when we used the prevalence of healthy lifestyle factors in NHANES for PAR calculation (eTable 3 in the Supplement).

**Table 2.** Healthy Lifestyle Factors Prior to the Pandemic and Risk of Post–COVID-19 Condition (PCC) Among Participants Who Reported a Positive SARS-CoV-2 Test During Follow-up, the Nurses’ Health Study II, 2015-2021

Characteristic	PCC/total COVID (n = 1981)	Relative risk (95% CI)		
		Model 1: Adjusted for age, race and ethnicity, and socioeconomic status^a^	Model 2: Further adjusted for health care worker status and history of chronic diseases^b^	Model 3: Model 2, mutually adjusted for other healthy lifestyle factors^c^
BMI				
<18.5	12/24	1.37 (0.77-2.45)	1.35 (0.76-2.43)	1.31 (0.73-2.34)
18.5-24.9	249/665	1 [Reference]	1 [Reference]	1 [Reference]
25-29.9	265/619	1.15 (0.97-1.37)	1.12 (0.94-1.33)	1.11 (0.93-1.32)
30-34.9	201/392	1.38 (1.14-1.66)	1.30 (1.07-1.58)	1.27 (1.04-1.55)
≥35	144/281	1.35 (1.09-1.67)	1.25 (1.00-1.56)	1.21 (0.96-1.52)
*P* for trend	NA	.001	.02	.04
Smoking				
Never	550/1309	1 [Reference]	1 [Reference]	1 [Reference]
Past	302/631	1.14 (0.99-1.31)	1.10 (0.96-1.27)	1.11 (0.96-1.29)
Current	19/41	1.09 (0.69-1.72)	1.04 (0.65-1.64)	1.02 (0.64-1.63)
*P* for trend	NA	.10	.23	.22
Diet^d^				
Q1	218/480	1 [Reference]	1 [Reference]	1 [Reference]
Q2	194/427	1.01 (0.83-1.22)	1.01 (0.83-1.22)	1.03 (0.85-1.26)
Q3	186/409	1.02 (0.84-1.24)	1.04 (0.85-1.27)	1.08 (0.88-1.32)
Q4	149/347	0.95 (0.77-1.18)	0.97 (0.79-1.21)	1.03 (0.83-1.28)
Q5	124/318	0.87 (0.69-1.09)	0.91 (0.72-1.14)	0.97 (0.77-1.23)
*P* for trend^e^	NA	.23	.44	.82
Alcohol consumption, g/d				
0	295/631	1.14 (0.94-1.37)	1.10 (0.92-1.33)	1.08 (0.89-1.30)
1.0–4.9	262/583	1.10 (0.91-1.32)	1.10 (0.91-1.33)	1.07 (0.88-1.29)
5.0–14.9	191/468	1 [Reference]	1 [Reference]	1 [Reference]
15.0–29.9	77/202	0.94 (0.72-1.23)	0.94 (0.72-1.23)	0.94 (0.72-1.23)
≥30	46/97	1.17 (0.85-1.62)	1.14 (0.83-1.58)	1.15 (0.83-1.59)
Moderate and vigorous physical activity, min/wk				
0–30	283/594	1.19 (1.01-1.41)	1.12 (0.94-1.33)	1.06 (0.88-1.27)
30–90	165/354	1.17 (0.96-1.42)	1.14 (0.93-1.38)	1.09 (0.89-1.33)
90–150	62/139	1.10 (0.83-1.45)	1.10 (0.83-1.45)	1.08 (0.81-1.43)
150–210	87/203	1.08 (0.85-1.37)	1.06 (0.83-1.35)	1.04 (0.81-1.32)
≥210	274/691	1 [Reference]	1 [Reference]	1 [Reference]
*P* for trend	NA	.03	.17	.41
Sleep duration, h/d				
≤5	75/146	1.25 (0.97-1.61)	1.18 (0.92-1.53)	1.18 (0.91-1.52)
6	245/480	1.25 (1.06-1.47)	1.21 (1.02-1.43)	1.21 (1.02-1.43)
7	325/794	1 [Reference]	1 [Reference]	1 [Reference]
8	177/450	0.96 (0.80-1.16)	0.97 (0.80-1.16)	0.96 (0.80-1.16)
9	39/90	1.07 (0.77-1.49)	1.01 (0.72-1.42)	0.99 (0.71-1.39)
≥10	10/21	1.14 (0.60-2.14)	1.11 (0.59-2.09)	1.07 (0.56-2.04)

Abbreviations: BMI, body mass index (calculated as weight in kilograms divided by height in meters squared); NA, not applicable; Q, quintile.

^a^ Model 1: Adjusted for age, race and ethnicity, partner’s education, census tract median household income, and census tract percentage population with bachelor’s degree or higher.

^b^ Model 2: Model 1 + health care working status and history of chronic obstructive pulmonary disease, cancer, diabetes, asthma, hypertension, high cholesterol, cardiovascular diseases, and inflammatory bowel disease.

^c^ Model 3: Model 2 + mutual adjustment for other lifestyle factors.

^d^ Diet measured as Alternative Healthy Eating Index (AHEI)–2010 score. Diet and alcohol intake were assessed in 2015; all other lifestyle factors were assessed in 2017.

^e^ *P* for trend analysis used indicator levels as a continuous variable.

**Table 3.** Dichotomized Healthy Lifestyle Factors Prior to the Pandemic and Risk of Post–COVID-19 Condition (PCC) Among Participants Who Reported a Positive SARS-CoV-2 Test During Follow-up, the Nurses’ Health Study II, 2015-2021

Healthy lifestyle factors	PCC/total COVID (n = 1981)	Relative risk^a^ (95% CI)	Relative risk^b^ (95% CI)	Population attributable risk percentage^b^ (95% CI)
BMI				
<18.5 or ≥25	622/1316	1 [Reference]	1 [Reference]	
18.5-24.9	249/665	0.79 (0.68-0.92)	0.85 (0.73-1.00)	10.3 (0.2-19.8)
Smoking				
Past or current	321/672	1 [Reference]	1 [Reference]	
Never	550/1309	0.88 (0.77-1.01)	0.91 (0.79-1.05)	3.0 (–1.8-8.0)
Diet^c^				
Lower 60%	598/1316	1 [Reference]	1 [Reference]	
Upper 40%	273/665	0.90 (0.78-1.05)	0.97 (0.83-1.12)	2.4 (–7.6-11.9)
Alcohol consumption, g/d				
<5 or >15	680/1513	1 [Reference]	1 [Reference]	
Moderate (5-15)	191/468	0.91 (0.77-1.07)	0.94 (0.80-1.11)	4.5 (–8.1-16.0)
Moderate/vigorous exercise, min/wk				
<150	510/1087	1 [Reference]	1 [Reference]	
≥150	397/965	0.86 (0.76-1.00)	0.94 (0.81-1.08)	3.4 (–4.5-11.2)
Sleep duration, h/d				
<7 or >9	330/647	1 [Reference]	1 [Reference]	
7-9	541/1334	0.80 (0.69-0.92)	0.83 (0.72-0.95)	6.6 (1.8-11.5)

Abbreviation: BMI, body mass index (calculated as weight in kilograms divided by height in meters squared).

^a^ Adjusted for age, race and ethnicity, partner’s education, census tract median household income, and census tract percentage population with bachelor’s degree or higher.

^b^ Adjusted for age; race and ethnicity; health care working status; partner’s education, census tract median household income; census tract percentage population with bachelor’s degree or higher; history of chronic obstructive pulmonary disease, cancer, diabetes, asthma, hypertension, high cholesterol, cardiovascular diseases, and inflammatory bowel disease; and other lifestyle factors included in the table. Reference group for relative risk calculation is all other COVID-19 cases not in low risk factor category as defined in table.

^c^ Diet measured as Alternative Healthy Eating Index (AHEI)–2010 score. Diet and alcohol intake were assessed in 2015; all other lifestyle factors were assessed in 2017.

Results were similar in sensitivity analyses defining PCC as having symptoms lasting at least 2 months or at least 4 months; including self-presumed COVID-19 cases; excluding persons who had been hospitalized due to COVID-19; using multiple imputation; restricting cases to participants with ongoing symptoms; excluding PCC cases with only psychological, cognitive, or neurological symptoms; and additionally adjusting for vaccination status (eTable 4 in the Supplement). A weaker association was observed when we excluded participants endorsing fatigue or excluded alcohol intake from healthy lifestyle factors (eTable 5 in the Supplement). The association did not differ by health care worker status (eTable 6 in the Supplement, *P* = .77 for interaction).

**Figure 2.**
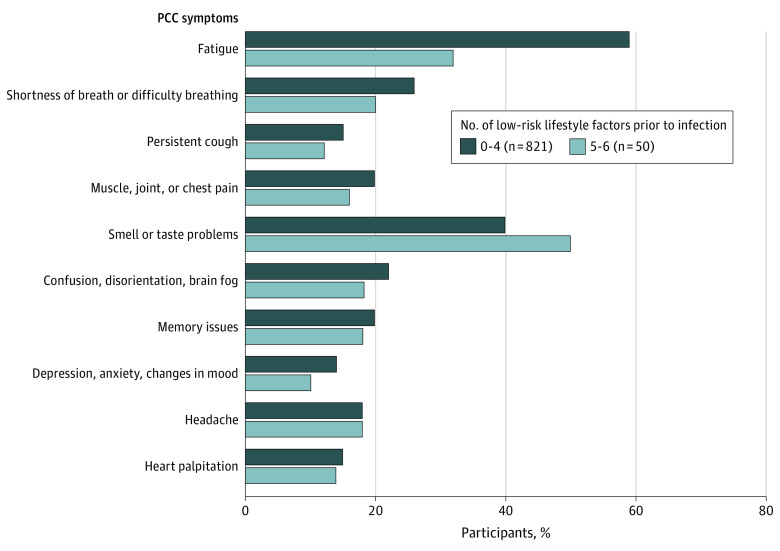
Post–COVID-19 Condition (PCC) Symptoms According to Number of Healthy Lifestyle Factors Prior to the Pandemic Among Persons Who Developed PCC, the Nurses’ Health Study II, 2015-2021 Healthy lifestyle factors include healthy body weight (body mass index, 18.5-24.9; calculated as weight in kilograms divided by height in meters squared), never smoking, at least 150 min/wk of moderate to vigorous physical activity, high diet quality (upper 40% of Alternative Healthy Eating Index [AHEI]–2010 score), moderate alcohol intake (5-15 g/d), and adequate sleep (7-9 h/d). Diet and alcohol intake were assessed in 2015; all other lifestyle factors were assessed in 2017.

Among participants who developed PCC, all COVID-19 symptoms were less prevalent in participants with higher healthy lifestyle scores, except for smell or taste problems and headache (0-4 factors, mean [SD] number of symptoms, 2.7 [1.8]; 5-6 factors, mean [SD] number of symptoms, 2.3 [1.6], Figure 2). Adherence to 5 to 6 vs 0 to 4 healthy lifestyle factors was associated with lower risk of daily life impairment due to PCC, although the CI was wide (RR, 0.70; 95% CI, 0.44-1.12).

## Discussion

In this prospective cohort study of women followed up for more than a year starting in April 2020, we found a beneficial dose-response association of a preinfection healthy lifestyle with risk of PCC, after accounting for sociodemographic factors and pre-existing conditions. Women endorsing 5 or 6 healthy lifestyle factors had approximately 50% lower risk of PCC than those without any healthy lifestyle factors. These associations were mainly driven by healthy body weight and adequate sleep. The PAR for all 6 healthy lifestyle factors in combination was 36.0%, indicating that, if these associations were causal, 36.0% of PCC cases would have been avoided if all participants had 5 or 6 healthy lifestyle factors prior to the pandemic.

Few studies have examined modifiable lifestyle factors preceding the pandemic as risk factors for PCC. Adherence to a healthy lifestyle has been associated with reduced risk of noncommunicable diseases and mortality,^19,33^ indicating a long-term health benefit. Specific to COVID-19, 2 prospective cohort studies using the UK Biobank (sample size approximately 400 000) found that a combination of lifestyle factors had a dose-response association with lower risk of COVID-19 hospitalization and mortality.^19,20^ Unhealthy lifestyle factors (smoking, physical inactivity, obesity, and alcohol drinking) in combination accounted for 51% of severe COVID-19 in the United Kingdom population.^20^ The associations between healthy lifestyle score and risk of severe COVID was partly mediated by low-grade inflammation (10% to 16%), as indicated by levels of C-reactive protein, although biomarkers were collected 10 years prior to infection.^20^ Our findings additionally identified a dose-response protective association of a healthy lifestyle against development of PCC, independent of pre-existing conditions and severity of acute phase disease.

While our results suggest that each of the 6 healthy lifestyle factors measured were broadly associated with a lower risk of PCC, in analyses mutually adjusted for all lifestyle factors and comorbidities, BMI and sleep were most strongly associated with lower risk of PCC. Several individual lifestyle factors have been associated with risk of long-term COVID symptoms or slow recovery from COVID-19, including obesity, smoking, unhealthy diet, and poor-quality sleep,^21,22,23,41,42,43^ although findings were not consistent, and no studies to our knowledge mutually adjusted for a range of lifestyle factors.^23^

Several biological mechanisms may explain the associations we observed. First, each unhealthy lifestyle factor we examined has been associated with increased risk of chronic inflammation, including findings from our cohort.^13,14,15,16,17,18,20,44,45,46,47,48,49,50,51,52,53^ Sustained systemic inflammation has been implicated in the development of PCC.^9^ Chronic inflammation may predispose individuals to excessive release of cytokines after infection, subsequently increasing risk for long-term complications in multiple organs.^54,55^ Second, these unhealthy lifestyle factors dysregulate adaptive autoimmunity, which has been found in individuals with PCC.^9,56,57,58,59,60^ Third, unhealthy lifestyle factors (obesity, smoking, physical inactivity, and excessive alcohol intake) predispose to blood clotting abnormalities, another pathophysiological change observed in persons with PCC.^61,62^ It has also been postulated that healthy lifestyle may benefit both innate and adaptive immune responses.^63,64,65^

Strengths of our study include a prospective design in which healthy lifestyle factors were assessed prior to the pandemic using validated instruments. In addition, incident SARS-CoV-2 infection, hospitalization due to COVID-19, COVID-19 vaccination, and PCC were ascertained during an active phase of the pandemic with monthly and quarterly follow-up over 19 months. Our study provides valuable population-based evidence for the association between healthy lifestyle and PCC.

### Limitations

Our study has several limitations. First, our cohort was comprised of middle-aged female nurses who were predominantly White, limiting generalizability. Moreover, because the incidence of PCC may differ by infecting strains,^66^ we cannot be certain that associations we found apply to PCC resulting from subsequent COVID-19 strains. In addition, we do not have information about multiple infections. Second, PCC information was not missing at random, which might have introduced bias. However, results were comparable in analyses using multiple imputation. Third, as SARS-CoV-2 infection and PCC were self-reported, misclassification may have occurred. Nevertheless, validity of self-reported health information is high in this cohort. Fourth, because PCC is still poorly understood, and there is no reference standard (or consensus) for the diagnosis of PCC, it is practically difficult to link symptoms with COVID-19 and to ascertain PCC cases.^67^ Finally, because asymptomatic cases are less likely to be detected, we likely underestimated the true prevalence of COVID-19 infections.

The findings for PAR should be interpreted with caution. The PAR relies on a causal interpretation of the association between healthy lifestyle factors and risk of PCC. As our study is observational, residual confounding likely remains, despite adjustment for multiple potential confounders. Although these healthy lifestyle factors are potentially modifiable, they are difficult to change;^68^ thus, eliminating this PAR may not be achievable. In addition, PAR is a population-specific calculation contingent on the prevalence of the exposures and their association with risk of disease; thus, the PAR calculated here may not apply to other populations. However, the prevalence of healthy lifestyle factors was comparable in female participants of the same age in the nationally representative NHANES cohort.

## Conclusions

The findings of this prospective cohort study indicate that adherence to a healthy lifestyle was associated with substantially reduced risk of developing PCC among individuals subsequently infected with SARS-CoV-2. If the associations we found were causal, among healthy lifestyle factors, maintaining a healthy weight and having adequate sleep duration may confer the greatest benefit for prevention of PCC. Future research should investigate whether implementing lifestyle interventions decreases risk of PCC or benefits persons with PCC or other chronic postinfection syndromes.

## Notes

Supplement 1. **eFigure 1.** Flowchart of COVID-19 substudy study design, the Nurses’ Health Study II (NHSII), 2015–2021 **eFigure 2.** Flowchart of study design, the Nurses’ Health Study II, 2015–2021 **eMethods.** Supplementary methods **eTable 1.** Age-standardized characteristics according to missingness of post–COVID-19 condition (PCC) information, the Nurses’ Health Study II, 2015–2021 **eTable 2.** Correlation (phi coefficient) among healthy lifestyle factors prior to the pandemic among participants reported a positive SARS-CoV-2 test during follow up, the Nurses’ Health Study II, 2015–2021, n=1981 **eTable 3.** Healthy lifestyle factors and population attributable risk percentage of post–COVID-19 condition by prevalence of risk factors among women ages 55 to 75 years in the US National Health and Nutrition Examination Survey (NHANES, 2013–2014) **eTable 4.** Healthy lifestyle factors prior to the pandemic and risk of post–COVID-19 condition (PCC) among participants who reported a positive SARS-CoV-2 test during follow up, the Nurses’ Health Study II, 2015–2021, n=1981 **eTable 5.** Healthy lifestyle factors excluding alcohol intake prior to the pandemic and risk of post–COVID-19 condition among participants who reported a positive SARS-CoV-2 test during follow up, the Nurses’ Health Study II, 2015–2021, n=1981 **eTable 6.** Healthy lifestyle factors prior to the pandemic and risk of post–COVID-19 condition among participants who reported a positive SARS-CoV-2 test during follow up, stratified by healthcare worker status, the Nurses’ Health Study II, 2015–2021, n=1981

Supplement 2.
Data Sharing Statement
