# Supplementary material for: Adherence to Healthy Lifestyle Prior to Infection and Risk of Post–COVID-19 Condition
Source: JAMA Intern Med. 2023 Feb 6;183(3):232–41. doi: 10.1001/jamainternmed.2022.6555 (PMC9989904; doi:10.1001/jamainternmed.2022.6555)
Supplement: Supplement 1. — eFigure 1. Flowchart of COVID-19 substudy study design, the Nurses’ Health Study II (NHSII), 2015–2021 eFigure 2. Flowchart of study design, the Nurses’ Health Study II, 2015–2021 eMethods. Supplementary methods eTable 1. Age-standardized characteristics according to missingness of post–COVID-19 condition (PCC) information, the Nurses’ Health Study II, 2015–2021 eTable 2. Correlation (phi coefficient) among healthy lifestyle factors prior to the pandemic among participants reported a positive SARS-CoV-2 test during follow up, the Nurses’ Health Study II, 2015–2021, n=1981 eTable 3. Healthy lifestyle factors and population attributable risk percentage of post–COVID-19 condition by prevalence of risk factors among women ages 55 to 75 years in the US National Health and Nutrition Examination Survey (NHANES, 2013–2014) eTable 4. Healthy lifestyle factors prior to the pandemic and risk of post–COVID-19 condition (PCC) among participants who reported a positive SARS-CoV-2 test during follow up, the Nurses’ Health Study II, 2015–2021, n=1981 eTable 5. Healthy lifestyle factors excluding alcohol intake prior to the pandemic and risk of post–COVID-19 condition among participants who reported a positive SARS-CoV-2 test during follow up, the Nurses’ Health Study II, 2015–2021, n=1981 eTable 6. Healthy lifestyle factors prior to the pandemic and risk of post–COVID-19 condition among participants who reported a positive SARS-CoV-2 test during follow up, stratified by healthcare worker status, the Nurses’ Health Study II, 2015–2021, n=1981 [file jamainternmed-e226555-s001.pdf]

Supplemental Online Content

Wang S, Li Y, Yue Y, et al. Adherence to healthy lifestyle prior to infection and risk of post–COVID-19 condition. *JAMA Intern Med.* Published online February 6, 2023. doi:10.1001/jamainternmed.2022.6555

eFigure 1. Flowchart of COVID-19 substudy study design, the Nurses' Health Study II (NHSII), 2015–2021

eFigure 2. Flowchart of study design, the Nurses' Health Study II, 2015–2021

eMethods. Supplementary methods

eTable 1. Age-standardized characteristics according to missingness of post–COVID-19 condition (PCC) information, the Nurses' Health Study II, 2015–2021

eTable 2. Correlation (phi coefficient) among healthy lifestyle factors prior to the pandemic among participants reported a positive SARS-CoV-2 test during follow up, the Nurses' Health Study II, 2015–2021, n=1981

eTable 3. Healthy lifestyle factors and population attributable risk percentage of post–COVID-19 condition by prevalence of risk factors among women ages 55 to 75 years in the US National Health and Nutrition Examination Survey (NHANES, 2013–2014)

eTable 4. Healthy lifestyle factors prior to the pandemic and risk of post–COVID-19 condition (PCC) among participants who reported a positive SARS-CoV-2 test during follow up, the Nurses' Health Study II, 2015–2021, n=1981

eTable 5. Healthy lifestyle factors excluding alcohol intake prior to the pandemic and risk of post–COVID-19 condition among participants who reported a positive SARS-CoV-2 test during follow up, the Nurses' Health Study II, 2015–2021, n=1981

eTable 6. Healthy lifestyle factors prior to the pandemic and risk of post–COVID-19 condition among participants who reported a positive SARS-CoV-2 test during follow up, stratified by healthcare worker status, the Nurses' Health Study II, 2015–2021, n=1981

This supplemental material has been provided by the authors to give readers additional information about their work.

© 2023 Wang S et al. *JAMA Internal Medicine.*

eFigure 1. Flowchart of COVID-19 substudy study design, the Nurses' Health Study II (NHSII), 2015–2021

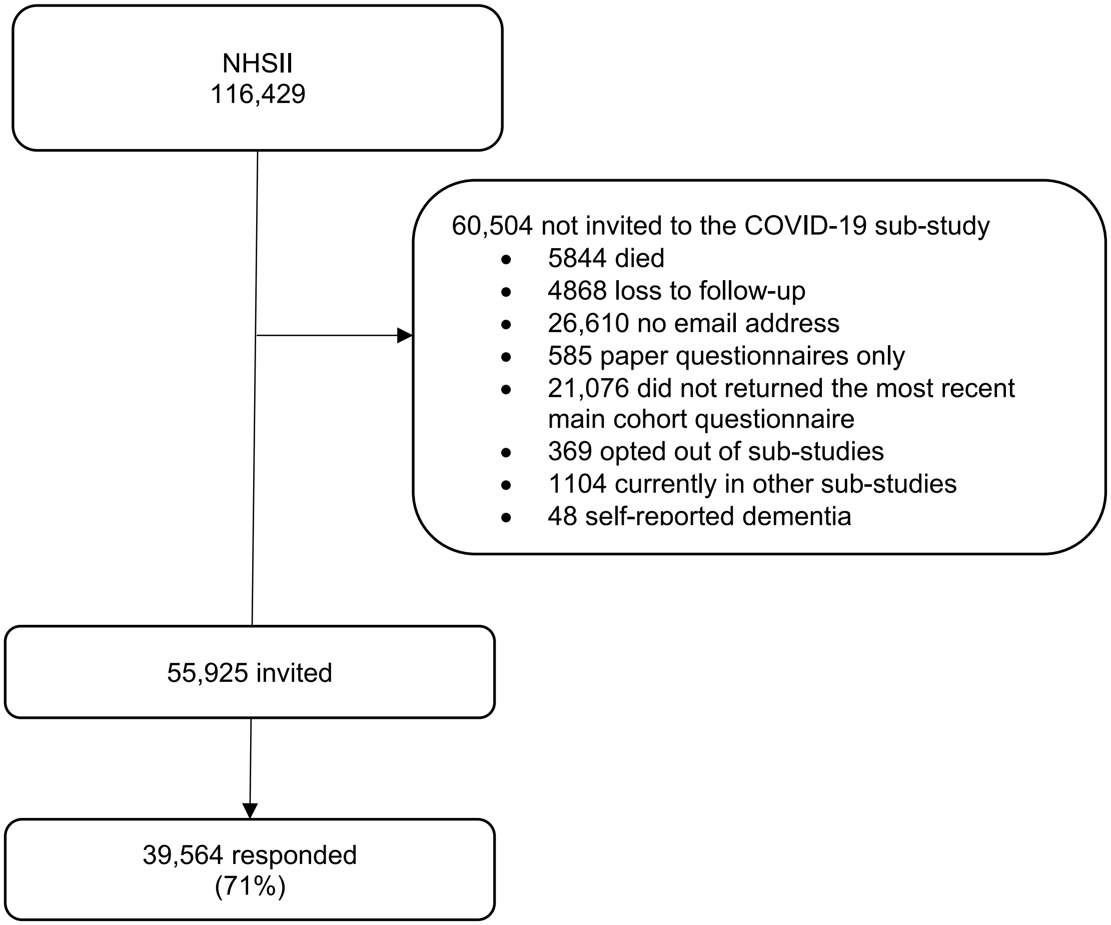

© 2023 Wang S et al. *JAMA Internal Medicine.*

eFigure 2. Flowchart of study design, the Nurses' Health Study II, 2015–2021

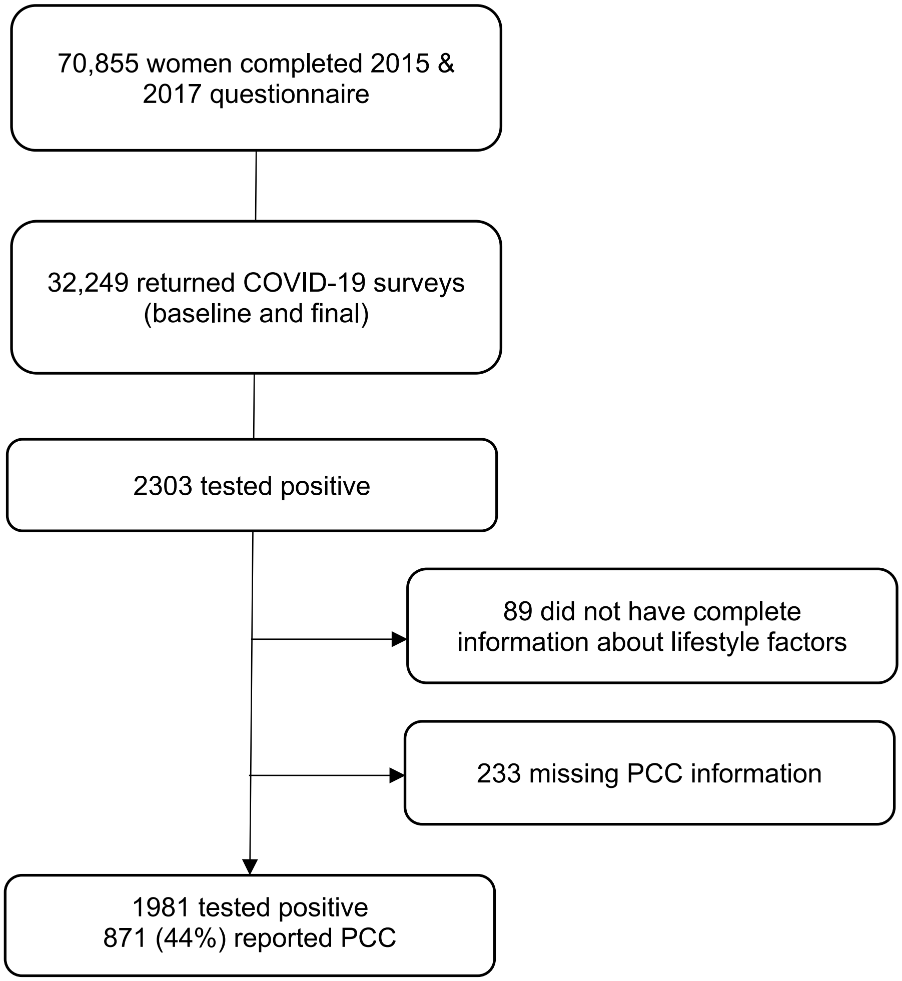

Abbreviation: PCC: Post–COVID-19 condition

Note. Lifestyle assessment: Diet and alcohol intake were assessed in 2015, all other lifestyle factors were assessed in 2017.

© 2023 Wang S et al. *JAMA Internal Medicine.*

eMethods

Long-term COVID-19 symptoms included fatigue, shortness of breath or difficulty breathing, persistent cough, muscle/joint/chest pain, smell/taste problems, confusion/disorientation/brain fog, memory issues, depression/anxiety/changes in mood, headache, intermittent fever, heart palpitations, rash/blisters/welts, mouth or tongue ulcers, or other symptoms.

We included physician-diagnosed chronic diseases with a lifetime cumulative incidence >2% in this cohort, including cancer, diabetes, high blood cholesterol, asthma, hypertension, chronic obstructive pulmonary disease, cardiovascular diseases (angina, myocardial infarction, coronary artery bypass graft, stroke, deep vein thrombosis), and inflammatory bowel disease (Crohn's disease and ulcerative colitis).

© 2023 Wang S et al. *JAMA Internal Medicine.*

eTable 1. Age-standardized characteristics according to missingness of post–COVID-19 condition (PCC) information, the Nurses' Health Study II, 2015–2021

		No. (%)	
		Missing PCC information	
		No	Yes
		n=1981	n=233
Age, y[a]		64.7 (4.6)	64.6 (4.6)
BMI, mean (SD), kg/m^2		28.4 (6.5)	27.1 (5.7)
Race, White[b]		1929 (97.4)	224 (96.1)
Partner's education ≤ high school		308 (15.6)	25 (10.7)
Census tract median household income, mean (SD), USD		65162.7 (24414.9)	70295 (27695.5)
Census tract % population with bachelor's degree or higher, mean (SD)		30.6 (17.2)	36.4 (20.0)
AHEI, mean (SD)		60.8 (12)	61.8 (12.5)
Alcohol, mean (SD), grams/day		7.2 (11.4)	8.1 (13.8)
Physical activity, min/week		228.6 (319.9)	236.9 (293.3)
Sleep, hours/day			
	≤5	146 (7.4)	28 (12.1)
	6	480 (24.2)	58 (24.9)
	7	794 (40.1)	84 (35.9)
	8	450 (22.7)	43 (18.5)
	9	90 (4.5)	20 (8.7)
	≥10	21 (1.1)	0 (0)
Smoking			
	Never	1309 (66.1)	156 (66.8)
	Past	631 (31.8)	68 (29.3)
	Current	41 (2.1)	9 (3.8)
Frontline health worker		849 (42.8)	134 (57.5)
**Lifetime history of comorbidities**			
High cholesterol		1201 (60.6)	134 (57.3)
Diabetes		232 (11.7)	15 (6.4)
Hypertension		851 (42.9)	88 (37.9)
Asthma		452 (22.8)	58 (24.9)
Cancer		415 (20.9)	58 (24.9)
Chronic obstructive pulmonary disease		98 (4.9)	8 (3.5)
Cardiovascular disease[c]		180 (9.1)	16 (7.0)
Inflammatory bowel disease		59 (3.0)	5 (2.2)

Abbreviations: AHEI, Alternative Healthy Eating Index; BMI, body mass index. Body mass index is calculated as weight in kilograms divided by height in meters squared.

[a] Not age-standardized.

[b] Race and ethnicity were self-reported at cohort entry. Values for categories other than White (American Indian/Alaska Native, Asian, Black or African American, Native Hawaiian or Pacific Islander, and other) are not presented because their number were small.

[c] Included angina, coronary artery bypass graft, myocardial infarction, stroke, and deep vein thrombosis.

© 2023 Wang S et al. *JAMA Internal Medicine.*

eTable 2. Correlation (phi coefficient) among healthy lifestyle factors prior to the pandemic among participants reported a positive SARS-CoV-2 test during follow up, the Nurses' Health Study II, 2015–2021, n=1981

	BMI	Smoking	Physical activity	Diet	Alcohol	Sleep
BMI	–					
Smoking	0.05	–				
Physical activity	0.24	0.00	–			
Diet	0.11	0.02	0.18	–		
Alcohol	0.08	-0.03	0.08	0.07	–	
Sleep	0.00	-0.02	0.03	0.08	0.07	–

Healthy lifestyle factors were coded as binary variables: healthy body weight (body mass index 18.5–24.9; calculated as weight in kilograms divided by height in meters squared), never smoking, at least 150 min/wk of moderate to vigorous physical activity, high diet quality (upper 40% of Alternative Healthy Eating Index [AHEI]-2010 score), moderate alcohol intake (5–15 g/d), and adequate sleep (7–9 h/d), versus those who did not meet these standards. Diet and alcohol intake were assessed in 2015, all other lifestyle factors were assessed in 2017.

© 2023 Wang S et al. *JAMA Internal Medicine.*

eTable 3. Healthy lifestyle factors and population attributable risk percentage of post–COVID-19 condition by prevalence of risk factors among women ages 55 to 75 years in the US National Health and Nutrition Examination Survey (NHANES, 2013–2014)

Healthy lifestyle factors	Nurses' Health Study II		NHANES	
	Percentage with healthy lifestyle factor (%)	Population attributable risk percentage (95% CI)	Percentage with healthy lifestyle factor (%)	Population attributable risk percentage (95% CI)
BMI, 18.5–24.9 kg/m^2	33.6	10.3 (0.2–19.8)	30.2	10.9 (0.2–20.7)
Smoking, never	66.1	3.0 (-1.8–8.0)	56.3	3.9 (-2.3–10.1)
Diet, upper 40% of alternative healthy eating index score	33.6	2.4 (-7.6–11.9)	40.0	2.2 (-6.9–11.0)
Alcohol consumption, moderate (5–15 grams/d)	23.6	4.5 (-8.1–16.0)	21.3	4.6 (-8.5–16.5)
Moderate/vigorous exercise, ≥150 min/wk	45.1	3.4 (-4.5–11.2)	38.2	3.9 (-5.1–12.5)
Sleep duration, 7–9 hours/d	67.3	6.6 (1.8–11.5)	64.0	7.1 (2.0–12.5)

Abbreviation: BMI, body mass index. Body mass index is calculated as weight in kilograms divided by height in meters squared.

Population attributable risk percentages were calculated based on RRs from Table 3.

© 2023 Wang S et al. *JAMA Internal Medicine.*

eTable 4. Healthy lifestyle factors prior to the pandemic and risk of post–COVID-19 condition (PCC) among participants who reported a positive SARS-CoV-2 test during follow up, the Nurses' Health Study II, 2015–2021, n=1981

Healthy lifestyle style score	Defined long COVID as symptoms lasting at least 2 months[a]	Defined long COVID as symptoms lasting at least 4 months[b]	Included 926 presumed SARS-CoV-2 infections without confirmatory test	Excluded 101 persons who had been hospitalized due to COVID-19	Multiple imputation of missing long COVID and lifestyle information[c]
			RR (95% CI)		
0	1.0 [reference]	1.0 [reference]	1.0 [reference]	1.0 [reference]	1.0 [reference]
1	0.94 (0.64–1.36)	0.98 (0.60–1.62)	0.94 (0.70–1.27)	0.90 (0.62–1.32)	0.93 (0.67–1.30)
2	0.85 (0.59–1.22)	0.83 (0.51–1.35)	0.85 (0.63–1.13)	0.83 (0.57–1.20)	0.86 (0.62–1.19)
3	0.77 (0.53–1.11)	0.74 (0.45–1.21)	0.81 (0.60–1.09)	0.80 (0.55–1.16)	0.81 (0.58–1.13)
4	0.70 (0.47–1.04)	0.66 (0.39–1.11)	0.84 (0.62–1.15)	0.75 (0.51–1.11)	0.75 (0.53–1.06)
5 or 6	0.52 (0.33–0.82)	0.58 (0.33–1.05)	0.64 (0.45–0.91)	0.51 (0.32–0.81)	0.53 (0.35–0.80)
P trend	0.001	0.079	0.060	0.001	0.003

(continued)

Healthy lifestyle style score	Defined long COVID as having ongoing symptoms (n cases=633)[d]	Excluded 157 persons reporting only psychological, cognitive, and neurological symptoms[e]	Excluded 497 persons reporting fatigue as one of their long COVID symptoms	Additionally adjusted for vaccination status at the time of infection	Main model, without adjustment for comorbidities
			RR (95% CI)		
0	1.0 [reference]	1.0 [reference]	1.0 [reference]	1.0 [reference]	1.0 [reference]
1	0.86 (0.58–1.26)	0.92 (0.63–1.35)	0.88 (0.48–1.62)	0.91 (0.64–1.29)	0.86 (0.61–1.22)
2	0.76 (0.52–1.10)	0.82 (0.56–1.19)	0.78 (0.43–1.41)	0.83 (0.59–1.17)	0.76 (0.54–1.06)
3	0.67 (0.46–0.98)	0.76 (0.52–1.12)	0.79 (0.44–1.43)	0.79 (0.56–1.12)	0.72 (0.51–1.01)
4	0.63 (0.42–0.94)	0.70 (0.47–1.04)	0.78 (0.43–1.44)	0.73 (0.51–1.06)	0.65 (0.45–0.92)
5 or 6	0.42 (0.26–0.69)	0.49 (0.31–0.79)	0.62 (0.32–1.20)	0.50 (0.32–0.77)	0.43 (0.28–0.66)
P trend	0.002	<0.001	0.15	<0.001	<0.001

© 2023 Wang S et al. *JAMA Internal Medicine.*

(continued)

Healthy lifestyle style score	Main model
	RR (95% CI)
0	1.0 [reference]
1	0.92 (0.65–1.31)
2	0.84 (0.60–1.18)
3	0.80 (0.57–1.14)
4	0.74 (0.51–1.07)
5 or 6	0.51 (0.33–0.78)
P trend	<0.001

Abbreviation: PAR: Population attributable risk percentage.

Healthy lifestyle factors include healthy body weight (body mass index 18.5–24.9; calculated as weight in kilograms divided by height in meters squared), never smoking, at least150 min/wk of moderate to vigorous physical activity, high diet quality (upper 40% of alternative healthy eating index [AHEI]-2010 score), moderate alcohol intake (5–15 g/d), and adequate sleep (7–9 h/d). Diet and alcohol intake were assessed in 2015, all other lifestyle factors were assessed in 2017.

Adjusted for age, race and ethnicity, healthcare worker status, partner's education, Census tract median household income, Census tract percentage population with bachelor's degree or higher, and history of chronic obstructive pulmonary disease, cancer, diabetes, asthma, hypertension, high cholesterol, cardiovascular diseases, and inflammatory bowel disease.

[a] Participants who had an initial infection within 2 months of the PCC assessment were excluded from the analysis (n=34). Non-cases were those who reported a positive test during follow up, and symptoms lasted for less than 2 months.

[b] Participants who had an initial infection within 4 months of the PCC assessment were excluded from the analysis (n=96). Non-cases were those who reported a positive test during follow up, and symptoms lasted for less than 4 months.

[c] Multiple imputation was performed with fully conditional specification using 20 imputed datasets.

[b] Participants who reported having had 4 weeks of symptoms but not having ongoing symptoms were excluded from the analysis (n=238).

[e] Definition: Participants with at least one of: smell/taste problems, shortness of breath or difficulty breathing, rash/blisters/welts, persistent cough, intermittent fever, muscle/chest pain, heart palpitations, mouth or tongue ulcers lasting at least 4 weeks; participants reporting only psychiatric and cognitive COVID-19-related symptoms lasting $\geq$ 4 weeks were excluded (e.g., only fatigue, confusion/disorientation/'brain fog', memory issues, depression/anxiety/changes in mood, and/or headache).

P trend analysis used indicator levels as a continuous variable

© 2023 Wang S et al. *JAMA Internal Medicine.*

eTable 5. Healthy lifestyle factors excluding alcohol intake prior to the pandemic and risk of post–COVID-19 condition among participants who reported a positive SARS-CoV-2 test during follow up, the Nurses' Health Study II, 2015–2021, n=1981

Healthy lifestyle style score	Multivariable model
	RR (95% CI)
0	1.0 [reference]
1	0.99 (0.71–1.36)
2	0.89 (0.65–1.22)
3	0.85 (0.62–1.18)
4 or 5	0.65 (0.45–0.92)
P trend	<0.001

PAR: Population attributable risk percentage.

Healthy lifestyle factors include healthy body weight (body mass index, 18.5–24.9; calculated as weight in kilograms divided by height in meters squared), never smoking, at least 150 min/wk of moderate to vigorous physical activity, high diet quality (upper 40% of alternative healthy eating index [AHEI]-2010 score), and adequate sleep (7–9 h/d). Diet was assessed in 2015, all other lifestyle factors were assessed in 2017.

Adjusted for age, race and ethnicity, healthcare worker status, partner's education, Census tract median household income, Census tract percentage population with bachelor's degree or higher, and history of chronic obstructive pulmonary disease, cancer, diabetes, asthma, hypertension, high cholesterol, cardiovascular diseases, and inflammatory bowel disease...

P trend analysis used indicator levels as a continuous variable.

© 2023 Wang S et al. *JAMA Internal Medicine.*

eTable 6. Healthy lifestyle factors prior to the pandemic and risk of post–COVID-19 condition among participants who reported a positive SARS-CoV-2 test during follow up, stratified by healthcare worker status, the Nurses' Health Study II, 2015–2021, n=1981

Healthy lifestyle style score	Active healthcare worker n=848	Non-active healthcare worker n=1133
	Multivariable model	
	RR (95% CI)	
0	1.0 [reference]	1.0 [reference]
1	0.85 (0.48–1.49)	0.99 (0.63–1.56)
2	0.86 (0.49–1.49)	0.84 (0.54–1.30)
3	0.76 (0.43–1.35)	0.84 (0.54–1.31)
4	0.70 (0.38–1.27)	0.78 (0.49–1.24)
5 or 6	0.57 (0.29–1.12)	0.47 (0.26–0.83)
P interaction	0.77	

Healthy lifestyle factors: Healthy lifestyle factors included healthy body weight (body mass index, 18.5–24.9; calculated as weight in kilograms divided by height in meters squared), never smoking, at least150 min/wk of moderate to vigorous physical activity, high diet quality (upper 40% of Alternative Healthy Eating Index [AHEI]-2010 score), and adequate sleep (7–9 h/d). Diet and alcohol intake were assessed in 2015, all other lifestyle factors were assessed in 2017.

Adjusted for age, race and ethnicity, partner's education, Census tract median household income, Census tract percentage population with bachelor's degree or higher, and history of chronic obstructive pulmonary disease, cancer, diabetes, asthma, hypertension, high cholesterol, cardiovascular diseases, and inflammatory bowel disease.cholesterol, cardiovascular diseases, and inflammatory bowel disease.

© 2023 Wang S et al. *JAMA Internal Medicine.*
