# Supplementary material for: Adherence to Healthy Lifestyle Prior to Infection and Risk of Post–COVID-19 Condition
Source: JAMA Intern Med. 2023 Feb 6;183(3):232–41. doi: 10.1001/jamainternmed.2022.6555 (PMC9989904; doi:10.1001/jamainternmed.2022.6555)
Supplement: Supplement 2. — Data Sharing Statement [file jamainternmed-e226555-s002.pdf]

Data Sharing Statement

Wang. Adherence to Healthy Lifestyle Prior to Infection and Risk of Post-COVID-19 Condition. *JAMA Intern Med*. Published February 06, 2023. doi:10.1001/jamainternmed.2022.6555

Data
Data available: No

Additional Information
Explanation for why data not available: Because of participant confidentiality and privacy concerns, data are available upon reasonable written request. According to standard controlled access procedure, applications to use NHS/NHSII/HPFS resources will be reviewed by our External Collaborators Committee for scientific aims, evaluation of the fit of the data for the proposed methodology, and verification that the proposed use meets the guidelines of the Ethics and Governance Framework and the consent that was provided by the participants. Investigators wishing to use NHS/NHSII/HPFS data are asked to submit a brief description of the proposed project (go to https://www.nurseshealthstudy.org/researchers (contact email: nhsaccess@channing.harvard.edu) and https://sites.sph.harvard.edu/hpfs/for-collaborators/ for details.
